# The KLF6 splice variant KLF6-SV1 promotes proliferation and invasion of non-small cell lung cancer by up-regultating PI3K-AKT signaling pathway

**DOI:** 10.7150/jca.34212

**Published:** 2019-08-29

**Authors:** Nan Zhang, Qian-Qian Yan, Lu Lu, Jing-Bo Shao, Zhi-Gang Sun

**Affiliations:** 1 Department of Oncology, Jinan Central Hospital Affiliated to Shandong University, Jinan 250012, People's Republic of China; 2 Shandong University; Department of Oncology, Jinan Central Hospital Affiliated to Shandong University, Jinan 250012, People's Republic of China; 3 Taishan Medical University; Department of Oncology, Jinan Central Hospital Affiliated to Shandong University, Shandong University, Jinan 250013, Shandong Province, China; 4 Weifang Medical University; Department of Thoracic Surgery, Jinan Central Hospital Affiliated to Shandong University, Jinan 250012, People's Republic of China; 5 Department of Thoracic Surgery, Jinan Central Hospital Affiliated to Shandong University, Shandong University, Jinan 250013, Shandong Province, China

**Keywords:** KLF6-SV1, splice variant, non-small cell lung cancer, siRNA, western blotting, proliferation, migration, invasion

## Abstract

Non-small cell lung cancer (NSCLC) is an aggressive type of lung malignancy. Most of the patients have poor prognosis. Increasing evidence has revealed an association between KLF6-SV1, known as an oncogenic splice variant of KLF6, and metastatic potential or poor prognosis in many cancers. We previously demonstrated the increased KLF6-SV1 expression in NSCLC samples. There was a significant association between increased expression of KLF6-SV1 with the pN and pTNM stages and poor survival in NSCLC patients. In the present study, we aimed to further investigate the functional role of KLF6-SV1 in the progression of NSCLC. SK-MES-1 cells were infected with Lenti-virus containing KLF6-SV1 to up-regulate its expression, and the small interfering RNA (siRNA) was designed to knock down KLF6-SV1 transcript level in A549 cells. CCK8, colony formation, wound-healing, and transwell assays were performed to examine cell proliferation, migration, and invasion respectively. Western blot assay was used to detect the expression or phosphorylation of related proteins. We found that *in vitro* silencing of KLF6-SV1 by siRNA inhibited A549 cell proliferation, migration, and invasion through changes in E-cadherin, N-cadherin, Vimentin, Snail1 and Snail2 expression. Furthermore, KLF6-SV1 isoform knockdown triggered caspase-dependent apoptosis of A549 cells through downregulation of the phosphatidylinositol 3-OH kinase (PI3K)/Akt signaling pathway and apoptosis-related protein expression. Overexpression of KLF6-SV1 transcript induced significant increase in proliferation, migration, invasion and changes in expression of related proteins. Our study support KLF6-SV1 might be an important player in modulating the growth, migration, invasion, and survival of NSCLC cells, and that silencing KLF6-SV1 siRNA has the potential to be a powerful gene therapy strategy for NSCLC.

## Introduction

Non-small cell lung cancer (NSCLC) persists as one of the leading causes of cancer related mortality. As one of the most widespread solid tumors in China, it is the primary cause of death from cancer 1, 2. More than 80% of lung cancer cases are due to NSCLC. By the time NSCLC has been detected, the cancer is advanced and the prognosis is poor 1. Even with multimodal therapy consisting of chemotherapy, radiation therapy, and surgery, there is a very low long-term disease-free survival rate in NSCLC patients 1, 3. Although previous studies have identified many tumor suppressor genes and oncogenes 4, crucial factors that contribute to the development of NSCLC have not been identified. Therefore, it is necessary to identify the molecular mechanism of carcinogenesis in order to develop new therapeutic targets.

Many recent reports have suggested that tumor suppressor gene *Kruppel-like factor 6* (*KLF6*; also referred to as *COPEB* and *ZF9* gene) is crucial for the development and progression of different cancers due to its involvement in the regulation of cancer cell proliferation, invasion, migration, and survival 5-8. So far, three alternatively spliced KLF6 isoforms have been identified, named KLF6-SV1, -SV2 and -SV3, respectively 9. KLF6-SV1 is known as the functionally inactive form of the KLF6 gene, and has been involved in numerous human solid cancers 6, including prostate 10, 11, gastric 12, glioma 13, nasopharyngeal 14, hepatocellular 8, 15-17, pancreatic 7, ovarian carcinomas 9 and et al. KLF6-SV1 is a new player in the promotion of tumor growth and dissemination. Upregulation of KLF6-SV1 has an association with a poor prognosis in cancers 10, 14, 16. Furthermore, KLF6-SV1 overexpression is associated with metastatic potential 18. Specific knockdown of KLF6-SV1 by small interfering RNA (siRNA) reduces tumor growth *in vitro* 6 and *in vivo* 10. Additionally, KLF6-SV1 has demonstrated anti-apoptotic activity independent of p53 status 19. Based on these results, KLF6-SV1 might be a novel anticancer target for in NSCLC therapy.

Although the KLF6-SV1 transcript is known to play a critical role in lung adenocarcinoma 20, 21, its specific involvement in NSCLC remains largely unknown. First, we demonstrated upregulation of KLF6-SV1 in NSCLC samples 22. We then sought to elucidate the molecular function of KLF6-SV1 transcripts. RNA interference (RNAi) was used for the production of specific silencing of KLF6-SV1 in the NSCLC cell line A549 (with strong KLF6-SV1 expression); we also used lentivirus overexpressing KLF6-SV1 to increase its protein in SK-MES-1 cells. We detected the cellular capacity for cell growth, migration, invasion, and survival of KLF6-SV1-silenced A549 and KLF6-SV1-overexpresing SK-MES-1 cells in vitro. Furthermore, possible downstream targets of KLF6-SV1 as well as KLF6- SV1 related signaling pathways were also investigated.

## Materials and Methods

### Cell culture

All the cell lines (H520, SK-MES-1, A549 and H1975) were obtained from the American Tissue Culture Collection (ATCC) and Institute of Cell Biology (Shanghai, China). The SK-MES-1 cell line was infected with Lenti-virus containing KLF6-SV1 according to the infection instructions. Lipofectamine 2000 (Invitrogen) was utilized for transient transfection of non-targeting control (5′-UAG CGA CUA AAC ACA UCA AUU) and specific SV1 siRNA (5′-CAG GGA AGG AGA AAA GCC UUU) in A549.

### Western Blot Analysis

In preparation for western blot analysis, TNE buffer was added into the cell lysates. The total protein concentration was measured using a BCA protein Assay kit (Beyotime, Nantong, Jiangsu, China) according to the manufacturer's instructions. Equivalent protein content (40~80 μg) were loaded and separated by SDS-PAGE, then transferred to PVDF membranes (EMD Millipore, Billerica, Massachusetts, USA). After blocking non-specific binding sites with a 5% non-fat milk solution for 1hours at room temperature, the PVDF membranes were incubated overnight at 4 °C with the following primary antibodies: KLF6-SV1 (Zymed, USA), p-AKT (Ser473), AKT, ERK1/2, p-ERK1/2 ( (Thr202/Tyr204, Cell Signaling Technologies, Danvers, MA, USA), E-cadherin, N-cadherin, Vimentin, Snail1, Snail2, Cyclin D1, cleaved caspase-3, Bcl-2, and Bax (Beyotime, China) were utilized to perform western blotting. The internal control was GAPDH (Beyotime, Nantong, Jiangsu, China). Probing with individual antibodies was followed by the visualization of antigen-antibody complexes with the enhanced chemiluminescence reagent Supersignal (Pierce Biotechnology, Inc. USA).

### CCK8 assay

*In vitro* cell growth was assessed at 24, 48, and 72 h, respectively. Cells were grown in monolayer culture to obtain 60% confluence followed by the addition of 0.25% trypsin. This was then plated at a density of 2000 cells/well into separate wells of a 96-well plate (Costar; USA). The culture medium consisted of DMEM-10% FBS supplemented with 100 IU/ml penicillin and 100 mg/ml streptomycin. The cells were incubated with CCK8 after 24, 48, and 72 h. Then, the enzyme- linked immunosorbent assay (ELISA) reader was used to measure the color intensity at 490 nm. The experiments were performed independently three times. The cell viability was expressed in relation to absorbance as A490 nm.

### Migration and invasion assays

We utilized 24-well Transwell chambers that had both upper and lower culture compartments separated by polycarbonate membranes with pores measuring 8-lm (Costar 3422; Corning) to evaluate motility and invasiveness of plasmid-transfected cells. Before cells were plated into the Transwells, DMEM-0.1% bovine serum albumin (BSA) was added into the upper chamber and incubated at 37°C for 1 h for saturation of non-specific binding sites to occur. Following incubation, this was removed and 5 X10^4^ cells suspended in 100 µl of DMEM- 0.1% BSA were placed into the top chamber. DMEM-10% FBS was added into the bottom chamber to act as a chemoattractant. Cells adhering to the lower membrane surface following migration through the pores were fixed with 3.7% paraformaldehyde, stained with 0.2% crystal violet, and then washed with phosphate buffered saline (PBS) three times. This was followed by dilution with 30% acetic acid, then cell number was counted via microscopy.

Similarly, Matrigel TM (Collaborative Biomedical Products, USA)-coated 24-well Transwell chambers were utilized to assess cell invasiveness. The concentration of Matrigel was 0.4 mg/ml. The protocol and analysis were similar to the migration assays. Both the migration and invasion assays of each cell group underwent three independent experiments, and the results were expressed as means ± SD.

## Results

### Expression of KLF6-SV1 in NSCLC cell lines

**Figure 1:**
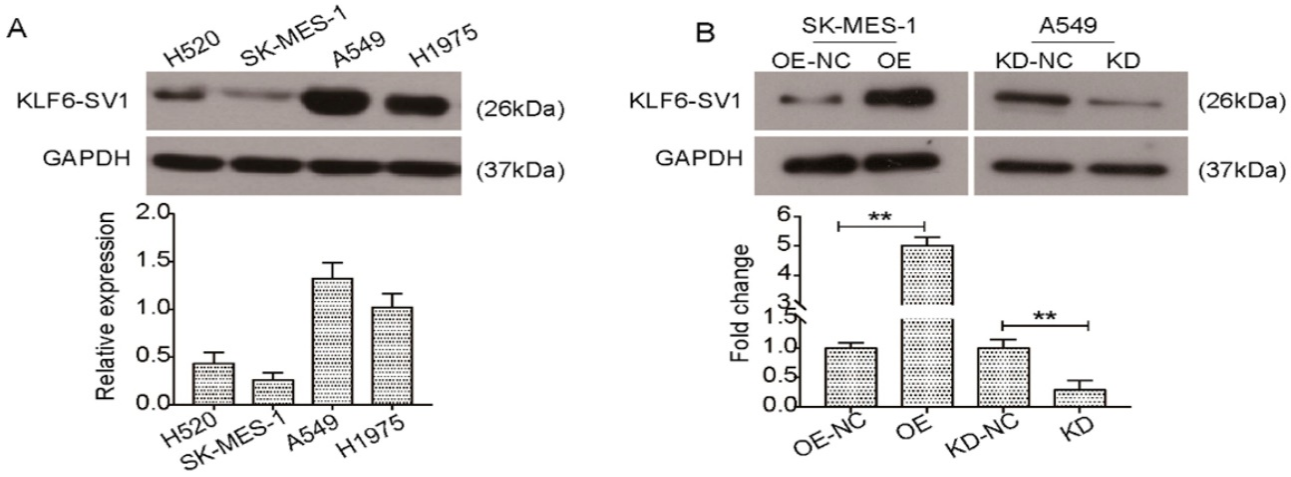
KLF6-SV1 was dysregulated in NSCLC cell lines. A. Western blot was used to detect the expression of KLF6-SV1 in different NSCLC cell lines including H520, SK-MES-1, A549, and H1975. B. SK-MES-1 cells were transfected with LV-KLF6-SV1 to upregulate the expression of KLF6-SV1; LV-NC was used as negative control. A549 cells were transfected with shRNA-KLF6-SV1 to knock down the expression of KLF6-SV1; scramble sequence was used as negative control. OE, cells were transfected with LV-KLF6-SV1 as the overexpression group (OE); OE-NC, cells were transfected with LV-NC as the negative control (NC); KD, cells were transfected with shRNA-KLF6-SV1 as the knockdown group (KD); KD-NC, cells were transfected with a scramble sequence as the negative control. Data are expressed as the mean ± SD from three independent experiments. **P<0.01 vs. the control group.

In previous studies, we reported that postoperative patients with non-small cell lung cancer contained the new prognostic biomarker KLF6-SV1 22. However, there is still a lack of understanding regarding the specific function of KLF6-SV1 in NSCLC. Here, we studied four well-characterized NSCLC cell lines, H520, SK-MES-1, A549, H1975, to determine KLF6-SV1 expression at protein levels. As shown in Fig. 1A, KLF6-SV1protein levels were much higher in the A549 and H1975 cells. In comparison, expression of KLF6-SV1 in the SK-MES-1 cell line was lower (Fig. 1A). To determine the biological relevance of these findings in the patient-derived samples, we developed the SK-MES-1 cell line overexpressing KLF6-SV1. Lentiviral infection was observed to cause 10-fold overexpression of KLF6-SV1 protein in comparison with empty virus-infected control cell lines (Fig. 1B). We used siRNA to suppress KLF6-SV1 expression in the A549 cell lines. As shown in Fig. 1B, si-SV1 transient transfection caused significant inhibition of KLF6-SV1 expression (KD). In si-SV1 transient-transfected cells, KLF6-SV1 expression was reduced by ~85% at 48 h compared with the NC control.

### KLF6-SV1 modulates cell proliferation, colony formation ability, migration, and invasion

**Figure 2:**
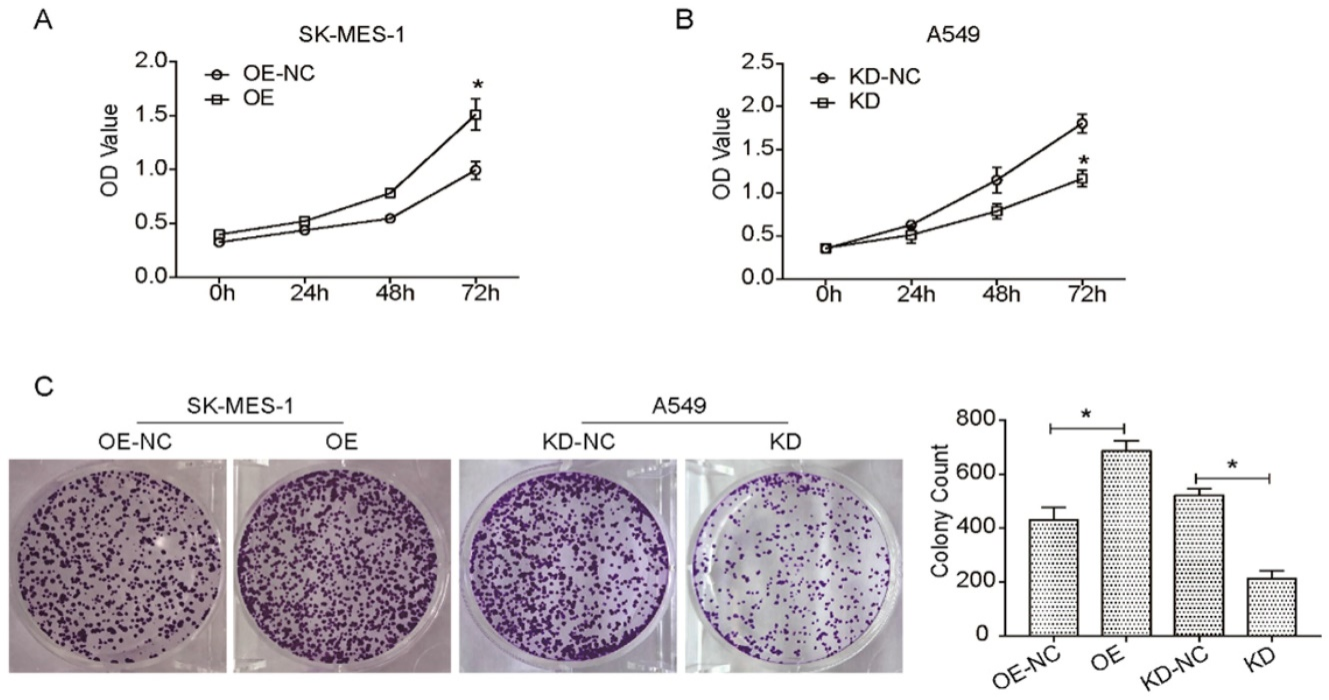
KLF6-SV1 increased cell viability and colony formation in NSCLC cells. Cells were transfected for 24h. A and B. CCK8 assay was used to assess cell viability in SK-MES-1 (A) and A549 (B) cells. C. After being transfected, cells were cultured for one week to form colonies in fresh medium. Data are expressed as the mean ± SD from three independent experiments. *P<0.05 vs. the control group.

Many reports have hypothesized that KLF6-SV1 overexpression could increase cell proliferation, migration, and invasion in numerous cancer cells 10, 13, 20-22. So, this study aimed to investigate the effect of the targeted KLF6-SV1 silencing through RNAi on the growth-related behavior of NSCLC cells. Cell viability in A549/KD-SV1 and SK-MES-1/OE-SV1 cells was detected by CCK8 assay. As shown in Fig. 2A, the cell viability of A549/KD-SV1 cells was reduced significantly compared with NC control cells. In contrast, the cell viability of SK-MES-1/OE-SV1 cells was increased significantly compared with control cells. Results from colony formation assay further supported the effect of KLF6-SV1 on the proliferation of NSCLC cells, in which upregulation of KLF6-SV1 significantly increased clonogenicity of SK-MES-1 cells, whereas downregulation of KLF6-SV1 significantly decreased clonogenicity of A549 cells (Fig. 2C).

**Figure 3:**
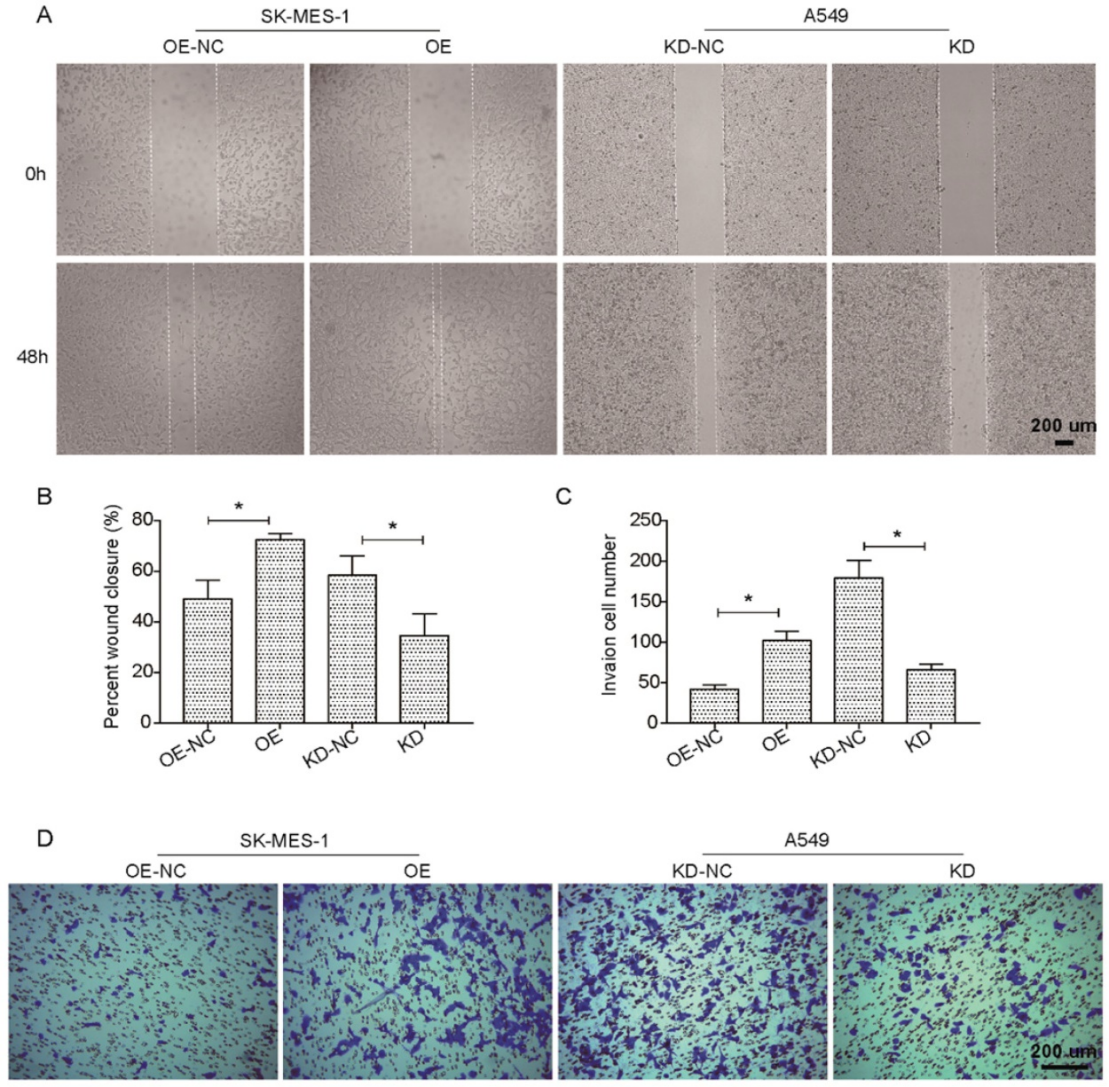
KLF6-SV1 increased cell migration in NSCLC cells in vitro. Cells were transfected for 24h. A and B. The wound-healing assay was used to evaluate cell migration ability of SK-MES-1 (left) and A549 (right) cells. C and D. Cell migration ability of SK-MES-1 (left) and A549 (right) cells was further assesses using Transwell migration assay. Data are expressed as the mean ± SD from three independent experiments. *P<0.05 vs. the control group.

We further investigated the potential effects of the suppression or overexpression of KLF6-SV1 on the ability of the *in vitro* migration and invasion of A549 and SK-MES-1 cells. Fig. 3A and B indicate that the migration ability of SK-MES-1 cells was significantly enhanced by overexpression of KLF6-SV1 compared to NC cells; in contrast, knocked-down of KLF6-SV1 resulted in inhibition of migration in A549 cells. In addition, we analyzed the function of KLF6- SV1 silencing or overexpression on the ability of NSCLC cells to invade into the Matrigel. The result is shown in Fig. 3C, D, A549/KD-SV1 showed 50% decreased invasiveness compared with NC-ctrl cells. On the other hand, SK-MES-1/OE-SV1 cells showed 50% increased invasiveness compared with NC-ctrl cells (Fig. 3C, D). The combination of data suggests that siRNA-induced silencing of KLF6-SV1 expression caused decreased proliferation, cell motility, and invasiveness of NSCLC cells in vitro; whereas, overexpression of KLF6-SV1 promoted the cell proliferation, motility, migration and invasion of SK-MES-1 cells.

### KLF6-SV1 alters the expression levels of EMT-related genes

**Figure 4:**
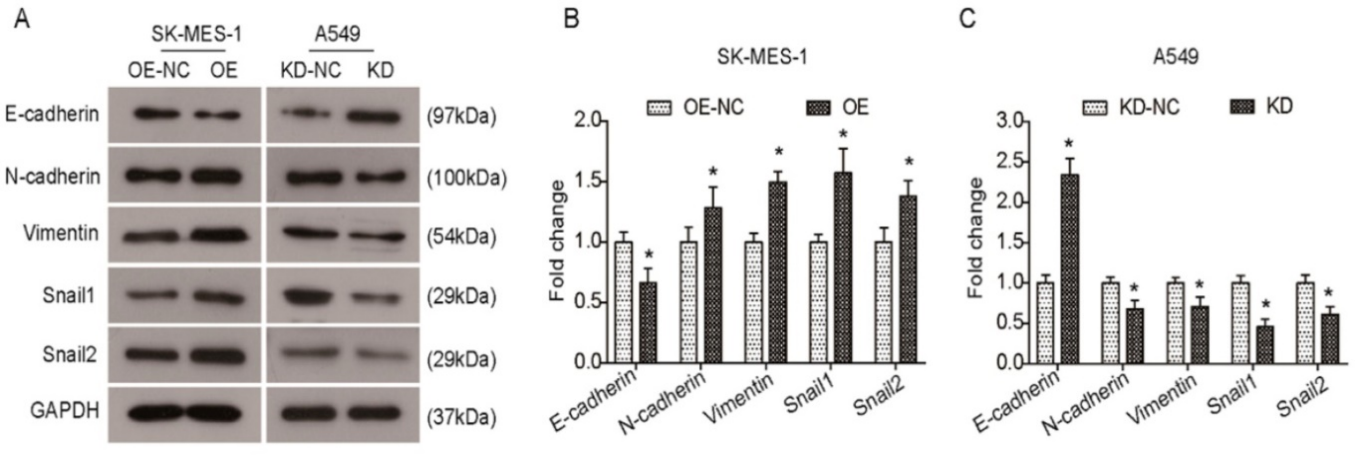
KLF6-SV1 promoted the EMT in NSCLC cells in vitro. A. After being transfected for 48h, changes in expression of EMT-related proteins were detected using western blot in SK-MES-1 (left) and A549 (right) cells. B and C. Quantitative analysis of the western blot results (Normalized to NC group). Data are expressed as the mean ± SD from three independent experiments. *P<0.05 vs. the control group.

Several Krüppel-like factors (KLFs) are involved in EMT and metastasis. Previous studies have shown that KLF6-SV1 overexpression can induce an EMT-like phenotype and results in marked cancer cell dissemination *in vivo*. Therefore, we sought to elucidate whether KLF6-SV1 has an effect on protein expression in lung cancer cells. As shown in Fig. 4A and B, there was a decrease in E-cadherin expression in the KLF6-SV1-upregulated clones of SK-MES-1 cells, while the expression of N-cadherin, Vimentin, Snail1, and Snail2 was significantly increased. In contrast, E-cadherin expression was upregulated in A549 cells after KLF6-SV1 siRNA transfection, while the expression of N-cadherin, Vimentin, Snail1, and Snail2 was downregulated (Fig. 4A and C). Therefore, these data indicate that the effect of KLF6-SV1 on tumor invasion might be mediated through regulation of EMT.

### KLF6-SV1 regulates the Akt signaling pathway in NSCLC cells

**Figure 5:**
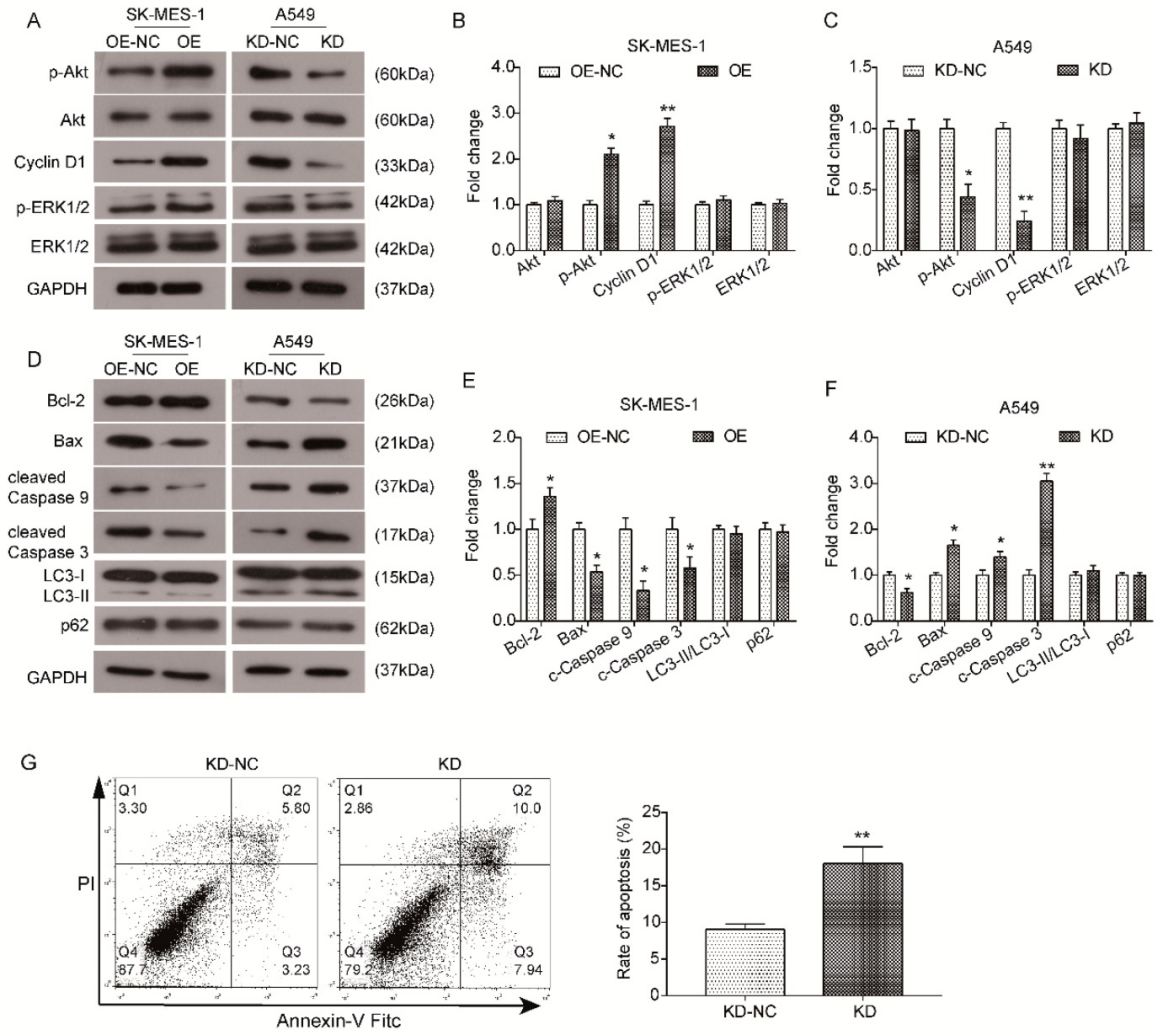
KLF6-SV1 upregulated the Akt signaling pathway in NSCLC cells. Cells were transfected for 48h. A. The changes in expression of Akt-related proteins were detected using western blot in SK-MES-1 (left) and A549 (right) cells. B and C. Quantitative analysis of the western blot results (Normalized to NC group). Data are expressed as the mean ± SD from three independent experiments. *P<0.05, **P<0.01 vs. the control group. D. The changes in expression of apoptosis-related proteins, Bcl-2, Bax, cleaved Caspase 9, and cleaved Caspase3, were detected using western blot in SK-MES-1 (left) and A549 (right) cells. E and F. Quantitative analysis of the western blot results (Normalized to NC group). Data are expressed as the mean ± SD from three independent experiments. *P<0.05, **P<0.01 vs. the control group.

The Akt signaling pathway has been proven to be a pivotal signaling pathway involved in tumorigenesis, growth and metastasis. Hence, we examined whether KLF6-SV1 affected the expression of key components in the Akt pathway to further investigate the functional mechanism underlying the oncogenic role of KLF6-SV1. As indicated by the results of western blot analysis, overexpression of KLF6-SV1 had no effect on the total level of Akt in SK-MES-1 cells but significantly promoted the phosphorylation of Akt (p-Akt, Ser473) but ERK1/2 (Fig. 5A and B) in comparison with NC cells. In correspondence, the expression of downstream protein Cyclin D1 was also upregulated in SK-MES-1/OE-SV1 cells (Fig. 5A and B). Similarly, the level of total Akt in si-KLF6-SV1 transfected A549 cells was upregulated as well (Fig. 5A and C). In contrast, the level of p-Akt was inhibited by depletion of KLF6-SV1 in A549 cells compared to NC cells, and the expression of Cyclin D1 was correspondingly downregulated (Fig. 5A and C). Furthermore, we utilized western blot analysis to examine the expression of apoptosis and autophagy-related proteins. As shown in Fig. 5D and E, the expression of anti-apoptotic protein Bcl-2 was increased in SK-MES-1/OE-SV1 cells, while the expression of pro-apoptotic proteins Bax, cleaved Caspase9 and cleaved Caspase3 was decreased (Fig. 5D and E). In contrast, the expression pattern of these proteins was reversed in KLF6-SV1-silenced A549 cells (Fig. 5D and F). The expression of two reliable autophagy marker LC3 and p62 was detected by western blot and no significant affect was detected upon KLF6-SV1 interference. These data suggest that KLF6-SV1 modulates cell proliferation in NSCLC cells possibly through regulation of apoptosis by regulating the Bcl-2/Bax axis and Caspase cascade. The ERK1/2 activity seems not affected by KLF-SV1 in NSCLC based on our present study. Further, we Collectively, the Akt signaling pathway may be involved in the functional mechanism underlying the oncogenic effect of KLF6-SV1 in NSCLC.

## Discussion

KLF6-SV1, a splice variant of KLF6, antagonizes the wild-type KLF6's tumor suppressive effects in many cellular processes, including proliferation, invasion and in vivo tumor growth. The expression of KLF6-SV1 has been reported to be up-regulated in many cancer types. Its overexpression was associated with poor survival in patients with multiple cancers.

It has been reported that high expression of KLF6-SV1 transcripts is associated with decreased survival in lung adenocarcinoma patients. Furthermore, KLF6-SV1 is a new anti-apoptotic protein in lung adenocarcinoma cell lines. Apoptosis was induced by siKLF6-SV1 alone or in combination with the chemotherapeutic drug cisplatin. Their reports highlight the key role of KLF6-SV1 transcripts in lung adenocarcinoma and show potential new therapeutic strategies for the treatment of lung cancer. We have previously reported an increase in KLF6-SV1 expression in NSCLC samples, and high expression of KLF6-SV1 has demonstrated a correlation with pN and pTNM stage and a poor survival rate in NSCLC patients. While, the detailed regulation of KLF6-SV1 in lung adenocarcinoma cell and whether KLF6-SV1 play roles in lung squamous cell carcinoma, are still not known. The aim of this study was to investigate and characterized the detailed functional role and the potential clinical relevance of KLF6-SV1 in both lung adenocarcinoma cell and squamous cell carcinoma.

In the present study, we also discovered differential expression of KLF6-SV1 in diverse differentiated NSCLC cell lines, indicating an additional role of KLF6-SV1 in the development of both lung adenocarcinoma cell and squamous cell carcinoma.

It has been demonstrated that abnormal KLF6-SV1 overexpression can result in significant changes in three important pathways controlling cancer growth including apoptosis, cellular proliferation, and metastasis. We designed siRNA to selectively inhibit KLF6-SV1 expression, then further investigated the effects of KLF6-SV1 siRNA on the proliferation, migration, invasion, and survival of lung adenocarcinoma cell line A549 in vitro. Following treatment with KLF6-SV1siRNA, the protein level of KLF6- SV1 was greatly decreased in A549 cells. We also constructed the expressing vector of KLF6-SV1 and detected the effects of KLF6-SV1 overexpression on cell function in SK-MES-1 cells (with low levels of KLF6-SV1), a typical squamous cell carcinoma cell line.

Our data revealed that in A549 cells, si-KLF6-SV1 could effectively suppress cell proliferation, migration, and invasion, which has not been reported previously in other cancers. In contrast, KLF6-SV1 overexpression demonstrated the opposite result in SK-MES-1 cells. Moreover, it is well known that KLF6-SV1 can modulate many EMT related-genes, including N-cadherin, E-cadherin, Snail-1and et al. In this study, we discovered that KLF6-SV1 can not only modulate the expression of N-cadherin, E-cadherin, Vimentin, Snail-1 and Snail-2, but can also reduce KLF6-SV1 downstream targets and mediators, including MMP-9 expression, and upregulated E-cadherin expression. This altered protein expression further impaired motility and invasion of cells. Additionally, KLF6-SV1 silencing could reduce activity of the PI3K-AKT pathway, which is a key pathway that is upregulated in human cancers 5, as well as reduce the expression of Cyclin D1.

Many studies have shown the involvement of KLF6-SV1 in cancer cell cycle and survival. Additionally, studies have shown that the targeted reduction of KLF6-SV1 using siRNA can induce apoptosis in lung cancer 10. Here, we further demonstrated that KLF6-SV1 depletion not only promoted apoptosis, but also inhibited migration and invasion in A549 cells. Furthermore, we reported KLF6-SV1 can also be functional in SK-MES-1 cells, which is a lung squamous cell carcinoma.

In conclusion, the data obtained in our report provides evidence of the mechanism of KLF6-SV1 involvement in the regulation of growth, migration, invasion, and survival of NSCLC. We reported that many key modulators of EMT, such as N-cadherin, E-cadherin, Snail-1 and et al were the downstream target of KLF6-SV1 in both both lung adenocarcinoma cell and squamous cell carcinoma. Additionally, we figured out that the PI3K-AKT signaling pathway was under the control of KLF6-SV1. Our study shows that KLF6-SV1 plays a very important role in non-small cell lung cancer, and this effect exists in both adenocarcinoma and squamous cell carcinoma. Intervention strategies for KLF6-SV1 may be a means of treating lung cancer.

## Abbreviations

NSCLC: Non-small cell lung cancer
KLF6: Kruppel-like factor 6
